# Women’s preferences for men’s facial masculinity are strongest under favorable ecological conditions

**DOI:** 10.1038/s41598-019-39350-8

**Published:** 2019-03-04

**Authors:** Urszula M. Marcinkowska, Markus J. Rantala, Anthony J. Lee, Mikhail V. Kozlov, Toivo Aavik, Huajian Cai, Jorge Contreras-Garduño, Oana A. David, Gwenaël Kaminski, Norman P. Li, Ike E. Onyishi, Keshav Prasai, Farid Pazhoohi, Pavol Prokop, Sandra L. Rosales Cardozo, Nicolle Sydney, Hirokazu Taniguchi, Indrikis Krams, Barnaby J. W. Dixson

**Affiliations:** 1 Jagiellonian University Medical College, Kraków, Poland; 2 Department of Biology, University of Turku, Turku, Finland; 3 Division of Psychology, University of Stirling, Glasgow, Scotland, United Kingdom; 4 Institute of Psychology, University of Tartu, Turku, Estonia; 5 Institute of Psychology, Chinese Academy of Sciences, Beijing, P. R. China; 6 ENES campus Morelia, National Autonomous University of Mexico, Mexico city, Mexico; 7 Department of Clinical Psychology and Psychotherapy, Babes-Bolyai University, Cluj-Napoca, Romania; 8 CLLE, Université de Toulouse, CNRS, UT2J, Toulouse, 31058, France; 9 Institut Universitaire de France, 103 boulevard Saint-Michel, 75005 Paris, France; 10 School of Social Sciences, Singapore Management University, Singapore, Singapore; 11 Department of Psychology, University of Nigeria, Nsukka, Nigeria; 12 Uniglobe H.S.S/College, Kathmandu, Nepal; 13 Department of Basic Psychology, School of Psychology, University of Minho, Braga, Portugal; 14 Department of Biology, Trnava University, Trnava, Slovakia; 15 Slovak Academy of Sciences, Bratislava, Slovakia; 16 Department of Psychology, University of Ibague, Department of Psychology, New York, Colombia; 17 Department of Zoology, Federal University of Parana, Curitiba, Brazil; 18 Department of Educational Psychology, Nagasaki University, Nagasaki, Japan; 19 Institute of Ecology and Earth Sciences, University of Tartu, Tartu, Estonia; 20 Department of Zoology and Animal Ecology, Faculty of Biology, University of Latvia, Riga, Latvia; 21 Department of Biotechnology, Daugavpils University, Daugavpils, Latvia; 22 School of Psychology, University of Queensland, Brisbane, Australia

## Abstract

The strength of sexual selection on secondary sexual traits varies depending on prevailing economic and ecological conditions. In humans, cross-cultural evidence suggests women’s preferences for men’s testosterone dependent masculine facial traits are stronger under conditions where health is compromised, male mortality rates are higher and economic development is higher. Here we use a sample of 4483 exclusively heterosexual women from 34 countries and employ mixed effects modelling to test how social, ecological and economic variables predict women’s facial masculinity preferences. We report women’s preferences for more masculine looking men are stronger in countries with higher sociosexuality and where national health indices and human development indices are higher, while no associations were found between preferences and indices of intra-sexual competition. Our results show that women’s preferences for masculine faces are stronger under conditions where offspring survival is higher and economic conditions are more favorable.

## Introduction

Sexual selection has shaped the evolution of male secondary sexual characteristics that communicate viability in many species^1^, including humans^2^. However, female preferences for males bearing attractive traits rarely converge on an optimal phenotype. This variation in preferences for sexually selected traits may occur in response to prevailing ecological, demographic and economic conditions^3^. Comparative studies suggest that men have a similar degree of sexually dimorphic secondary sexual trait development as nonhuman primate species with polygynous mating systems^4^, large social group sizes and complex multi-level social organizations^5^. This suggests that sexual selection via female choice and male-male competition has shaped the evolution of masculine traits in men^2^.

Men’s secondary sexual traits first appear during adolescence due to androgen exposure and are fully developed by young adulthood^2^. Physical masculinity may provide relevant information to potential mates and same-sex rivals regarding male reproductive maturity, underlying health, formidability, and social status^2^. One of the best studied sexually dimorphic traits is facial masculinity, which includes jaw size, brow ridge prominence, and robustness of the midface^6^. Facial masculinity is positively associated with some aspects of men’s health^7,8^ and disease resistance^9^. However, some research has not found this association^10^ or reported that a combination of facial masculinity and facial muscularity determines men’s immune response and women’s perceptions of men’s facial attractiveness^11^. Alternatively, facial masculinity may communicate more direct benefits like resource provisioning ability and protection^2,12^. More masculine looking men tend to have more muscular physiques^13,14^ and hence greater physical strength^12^, health^15^ and competitive ability^16^. Facial masculinity is also associated with behavioral dominance^17^, an open sociosexual orientation^18^, and higher social rank in same-sex dominance hierarchies^19^. Taken together, there is some evidence that women’s preferences for facial masculinity reflect selection for indirect (i.e. genetic) and direct benefits.

While several studies have reported masculine men to have higher mating success than less masculine men^19,20^, in many cases women’s preferences for facial masculinity are equivocal^21^ or less masculine-looking men are judged as more attractive than more masculine-looking men^22–24^. Variation in women’s preferences may be explained by the costs associated between masculinity and social traits^25,26^, as masculine looking men are perceived to be less warm, kind, and less paternally investing^24,27^. Further, masculine men state higher preferences for short-term than long-term relationships^18,28^, engage in more short-term relationships than their less masculine peers^28^ and women accurately assigned the likelihood of sexual infidelity from masculine facial shape in static photographs^29^. Thus, variation in preferences for masculine men may reflect choices for more prosocial partners and nurturing fathers over possible indirect and direct benefits associated with masculine facial traits^30,31^.

Evolutionary mating strategies theories propose that women overlook the costs of selecting less paternally investing masculine mates to secure benefits associated with phenotypic masculinity that could enhance offspring fitness^32^. Indeed, preferences for facial masculinity were highest among women living in countries and states within the U.S.A with lower national health^33,34^ and higher levels of pathogens^35^. These findings are bolstered by experimental studies reporting that exposure to cues of pathogens result in higher preferences for facial masculinity (^36^, but see ^37^). This suggests that any social costs of selecting masculine partners may be circumvented under conditions where potential indirect benefits may be realised. However, reanalysis of the data from DeBruine *et al*.^33^ reported that women’s preferences for facial masculinity were strongest in countries with high homicide rates and income inequality (indices of male intra-sexual competition) rather than reduced national health^38^,^39^. Experimental studies also demonstrate that women state higher preferences for facial masculinity following exposure to cues of male-male violence^40^. It is therefore possible that women’s preferences for masculine mates are stronger under conditions favoring direct material contributions rather than indirect genetic benefits.

Variation in women’s masculinity preferences could also emerge as a consequence of the density of available mates within the mating pool, whereby the saliency of masculine ornaments as attractive signals is stronger in larger and more complex social systems^40,41^. Recent research has found support for this hypothesis, as women’s preferences for masculine facial traits are stronger in larger cities with greater income disparity^40^ and in cultures with high human developmental indices^41^. This could indicate that under conditions where individuals assess the value of potential mates and rivals from among many anonymous conspecifics, masculine facial features become important as cues of distinctiveness^41^. Experimental evidence also shows causative effects of frequency dependence on women’s mate preferences, whereby masculine traits are more attractive when they are rare than when they are common^42^. However, whether women’s mate preferences for masculine characters are stronger when indirect or direct benefits may be prioritized, or whether economic development best explains variation, remains to be determined.

In addition to demographic variables, individual differences in mating strategies may contribute to the maintenance of variation in women’s preferences for facially masculine mates. Thus, women’s preferences for masculine traits may become stronger under conditions where the costs of lower paternal investment are reduced^32^. Indeed, women’s preferences for masculine faces, bodies, voices and odors are stronger when considering short-term than long-term mates^43^. Preferences for masculine partners as short-term mates may be strongest at the peri-ovulatory phase of the menstrual cycle, when any indirect benefits to offspring health and survivability could be acquired^32^. Initial research reported that women’s preferences for masculine physical, vocal, olfactory and behavioral traits were strongest at the peri-ovulatory phase of the menstrual cycle^44^. However, the majority of these studies employed indirect counting methods from questionnaires to ascertain women’s current fertility, which have been shown to be highly inaccurate compared measuring hormones^45^. Further, between-subject designs with small sample sizes were also methodological concerns in early studies of menstrual cycle effects on women’s mate preferences^46^. While some studies that measured women’s hormones to characterize current fertility reported women’s preferences for masculine characters were stronger when conception is more likely^47^, several recent studies have found no change in mate preferences among women at the peri-ovulatory phase for muscularity^48,49^, facial masculinity^23,49,50^, beards^23,51^, vocal masculinity^52,53^ and facial symmetry^49^. Thus, the extent to which ovulatory cycle shifts are associated with women’s mate preferences may have been overestimated in past research due to imprecise methodologies and small sample sizes^54^.

Individual differences in sociosexuality (SOI) are also associated with variation in women’s mate preferences. SOI refers to the desires, propensity to engage in and attitudes towards short-term, uncommitted and long-term, committed sexual partners^55^. More sexually open or unrestricted people report high scores for sexual openness, more sexual partners and may not place high importance on sexual monogamy. By contrast, people with a more restricted sociosexuality have fewer sexual partners and place greater importance on monogamy, love and fidelity^55,56^. SOI varies both within and between cultures in ways that conform to mating strategies theories. For example, willingness to participate in uncommitted sexual relationships is lower among people living under prevailing conditions of high parasite loads^57,58^ and where adult and infant survivability is low^59^. These patterns in sexual openness may reflect disease avoidance^57^ or direct benefits associated with bi-parental care under conditions where infant survival may be highly compromised^59^. As women’s preferences for facial masculinity may also reflect trade-offs between genetic quality and paternal investment^33^, individual differences and cross-cultural variation in SOI may predict women’s preferences for facial masculinity. While sociosexually open women state higher preferences for facial masculinity^60–62^, whether or not variation in women’s SOI and explains preferences for facial masculinity cross-culturally remains to be more fully explored^33^.

An important limitation in drawing conclusions across previous cross-cultural studies of women’s facial masculinity preferences concerns the statistical approaches employed^63^. Some cross-cultural research used aggregation at the national level to make claims relating to individual differences in mate choice^33,38^, which may inflate individual-level differences in preferences^63^. Further, cross-national demographic variables concerning health, violence, and economic stability are highly inter-correlated, such that unique variance attributable to specific demographic variables is challenging to expose when using aggregate national-level data^63^. The current study addresses these issues using facial masculinity preferences collected among 4483 heterosexual women from 34 countries. We employed mixed effects modelling to quantify individual-level preferences with national indices that capture health, pathogen prevalence, economic development and homicide rate. To address issues of multicollinearity among national demographic variables, we used an Independent Factors Analysis (IFA) to reduce number of country-level predictors. IFA is similar to PCA but allows factors to be correlated (i.e., not orthogonal), which can then be used as predictors in the fixed effects model. We used these data to test whether women’s preferences for facial masculinity were stronger under conditions where survival is compromised via pathogen prevalence and selecting a masculine mate may indirectly enhance offspring survival^33–35^. We also tested two alternative hypotheses that women’s facial masculinity preferences may be stronger under conditions where male survival is impacted by homicide rates^38^ or under conditions of high population density and wealth^41^. Finally, we asked whether variation in women’s sexual openness predicts their facial masculinity preferences.

## Material and Methods

### Facial masculinity

Photographs of 20 Caucasian men’s faces (aged 18–24 years) were taken under standard conditions. Facial masculinity was manipulated in PSYCHOMORPH program on a femininity–masculinity scale by adding or subtracting 50% of the linear difference between a 40 adult-male composite (average masculine face) and a 40 adult-female composite (average feminine face). This approach has been used in past studies to manipulate sexual dimorphism in facial morphology^33,64,65^. Twenty pairs of facial stimuli that differed only in facial shape within each pair were created. Presentation of the pairs of stimuli was fully randomized and the position of masculinized face relative to the feminized face on the right or left side of the screen was also fully randomized.

### Procedure

Participants completed an online survey, which began by measuring facial masculinity preferences using a two-alternative forced-choice (2AFC) experiment in which they selected from the 20 pairs of faces the face they considered to be most sexually attractive, following previous studies^66,67^. We chose 20 trials in a paired choice paradigm based on past studies^33,66,67^, methodological recommendations^68^ and simulations showing that 20 paired trials are sufficiently powered and including more trials has diminishing returns for augmenting statistical power against a 50% (i.e. 0.5) chance level^69^. Each pair of stimuli contained two versions of the same face, one that had been manipulated to be more facially masculinised, while the other more facially feminised. After completing the 2AFC part of the survey, participants also completed a short socio-demographic survey (including questions on ethnicity of parents, sexual orientation, pregnancies, lactation, and hormonal contraception use) and the Sociosexual Orientation Inventory Revised (SOIR; ^70^).

### Cross-national data

To test our predictions relating to demographic variation and women’s preferences for male facial masculinity, we sourced eleven national level data from online databases. These were chosen because they have previously been used in cross-national analyses of women’s facial masculinity preferences. First, six statistics were collected from The World Bank Database; these included women’s fertility rate (the average number of children per woman across her lifetime assuming she survives to a reproductive age in a given country), homicide rate (the number of unlawful deaths per year per 10,000 individuals), proportion of the population urbanised (the percentage of the total population of a given country living in urban areas), gross domestic product (GDP; the total market value of goods and services of a given country), overall mortality rate (the total number of deaths per 1,000 individuals per year), and the Gini coefficient (a measure of income of wealth distribution in a country). Years lost to communicable disease and adult life expectancy (the average lifespan in years in a given country) was collected from The World Health Organisation Database. The Human Development Index (HDI; a composite statistic where a country with higher lifespan, education level, and GDP per capita would score higher), and the Gender Inequality Index (GII; where countries with higher gender inequality would score higher) was collected from The United Nations Database. Finally, historical disease prevalence (9-items) was taken from Murray & Schaller^71^. Missing data for any country on any statistic was replaced with the mean of that statistic for the sample; this included three countries on the Gini coefficient (Singapore, New Zealand, and Saudi Arabia), and one country each on the GII and historical disease prevalence (Nigeria and Brazil respectively).

One issue with cross-national studies is that demographic variables reflecting health, violence, and economic stability are highly inter-correlated. To address this issue, we used Independent Factors Analysis (IFA) to reduce the 11 of country-level predictors to two factors. Factor loadings of each country-level statistic from the IFA are reported in Table 1. Factor 1 appeared to capture country health and development level and explained 41% of total variance in country-level statistics. Factor 2 appeared to capture country inequality and explained 26% of total variance. Both factors were reverse-coded, such that higher scores on Factor 1 represented better health/development, while higher scores on Factor 2 indicated greater equality. The two factors were positively (though non-significantly) correlated (*r* = 0.26, *p* = 0.142).

**Table 1:** Factor loadings of each country-level statistic from the IFA.

	Factor 1: Health/Development	Factor 2: Inequality
Life Expectancy at Birth	**−0.95**	−0.00
Human Development Index (HDI)	**−0.91**	−0.19
Years Lost to Disease	**0.87**	0.11
Urbanisation	**−0.82**	0.37
Fertility Rate	**0.76**	0.03
Historical Pathogen Prevalence (9-items)	**0.51**	0.36
GII	0.41	**0.56**
Mortality Rate	0.34	**−0.74**
Homicide Rate	0.16	**0.73**
GINI	0.15	**0.88**
GDP	−0.27	0.26

Note: Factors were reverse-coded in subsequent analyses.

### Participants

Participants were recruited via University web pages and websites or within Universities through information boards advertising the online address^67,72^. Data were collected from 7,739 female participants (*M* = 27.23 years, *SD* = 8.89 years) from 92 countries. Participants completed an online survey that had been translated into the national language of each country by research collaborators who spoke the national language fluently. Following previous research^33^, participants under 18 years (N = 101) or over 40 years (N = 699) of age were removed as peri-pubertal and postmenopausal women judge masculine faces differently^73–75^. Preferences for masculine traits also vary with sexual orientation^76–80^. Thus, participants who did not report being exclusively heterosexual were also removed (N = 1510). Finally, we removed participants who did not complete the SOIR (N = 857), and participants from countries with less than 10 participants (N = 89), resulting in a final sample of 4483 participants (*M* = 25.21 years, *SD* = 5.44 years) from 34 countries. All participants completed the full 20 trials and the average number of participants per country was 131.86 (SD = 200.35; for full breakdown, see the ESM). From the final sample, 2,486 women reported being in a stable relationship, 1,625 reported not being in a stable relationship, while 372 either did not report relationship status or indicated that it was “difficult to say”. Relationship status was not associated with preference for masculinity (see ESM for full analysis details).

### Statistical Analysis

To test whether country-level factors influenced women’s preference for facial masculinity, we analysed the data using a Binomial Mixed Effect Modelling at the level of the participant-trial interaction with the outcome variable being whether the masculinised or feminised face was chosen as more attractive (coded 1 and 0 respectively). Fixed effects included participant’s age, participant’s SOI, and the two factors from the IFA. Continuous predictors were z-standardised at the appropriate level before being entered into the models. Random intercepts included participant id, country, and face id, which accounts for the potential non-independence of observations made by the same participant, participants in the same country, and of the same stimuli across participants. An additional random effect of geographical region was included to account for potential non-independence between countries based on geographical location (e.g., similar climate, cultural history, see^81^). Data for geographical region were taken from the World Bank’s “Country and Lending Groups” classifications. Random slopes were specified maximally according to Barr *et al*.^82^. To test whether country-level factors influenced women’s SOI scores, we ran an additional Linear Mixed Effect Model at the participant-level with SOI score as the outcome variable. Fixed effects for participant age and both factors of the IFA, with region and country specified as random intercept. All analyses were conducted using R and the lme4^83^ and lmerTest^84^ packages. Full model specifications and results (including random effects) are supplied in the ESM.

### Ethical approval

Project was approved by the Ethics in Research Committee of Daugavpils University, Latvia and were their guidelines. All participants provided informed consent before participating in the study.

## Results

### Facial masculinity preferences

**Figure 1:**
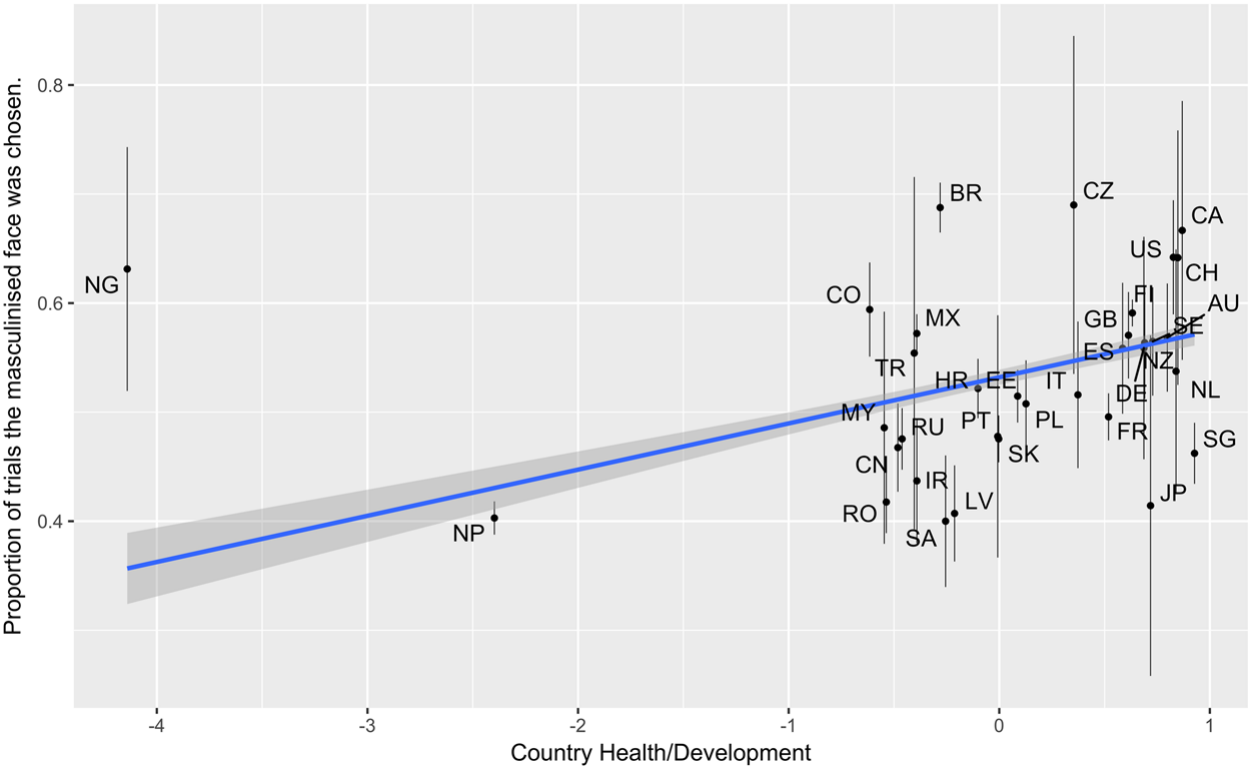
The association between country health/development factor and masculinity preference. Points represent the mean proportion that the masculine face was chosen for individuals in each country, with bars around points representing the 95% confidence interval of the mean. The regression line shows the regression line between average proportion of trials in which masculine faces were chosen for each country and country health/development. The shaded areas around the regression line are 95% confidence intervals. The country abbreviations in the figure are as follows: AU = Australia; BR = Brazil; CA = Canada; CH = Switzerland; CN = China; CO = Colombia; CZ = Czechia; DE = Germany; EE = Estonia; ES = Spain; FI = Finland; FR = France; GB = United Kingdom of Great Britain and Northern Ireland; HR = Croatia; IR = Iran; IT = Italy; JP = Japan; MX = Mexico; MY = Malaysia; NG = Nigeria; NL = Netherlands; NP = Nepal; NZ = New Zealand; LV = Latvia; PL = Poland; PT = Portugal; RO = Romania; RU = Russia; SA = Saudi Arabia; SE = Sweden; SG = Singapore; SK = Slovakia; TR = Turkey; US = United States of America.

The fixed effects from the model predicting women’s preference for facial masculinity are reported in Table 2. The intercept was negative (though non-significant), suggesting that women overall slightly preferred facial femininity. Participants’ age positively predicted preferences for facial masculinity, suggesting that older participants were more likely to prefer facial masculinity. Similarly, there was a significant, positive relationship between participant SOI and facial masculinity preferences, such that sociosexually unrestricted women preferred more facially masculine men. There was also a significant, positive association between the Health/Development factor and facial masculinity preference, such that as the health of a nation and/or development increased, preference for facial masculinity increased (Fig. 1). There was no significant association between the Inequality factor and facial masculinity preference.

**Table 2:** The fixed effects from the model predicting women’s preference for facial masculinity.

	Estimate (Std. Error)	*z*-value	*p*-value
Intercept	−0.49 (0.45)	−1.09	0.277
Participant Age	0.04 (0.01)	6.21	<0.001
Participant SOI	0.11 (0.03)	3.85	<0.001
Country Health/Development Factor	0.29 (0.14)	2.01	0.045
Country Inequality Factor	−0.05 (0.12)	−0.40	0.692

### SOI

**Figure 2:**
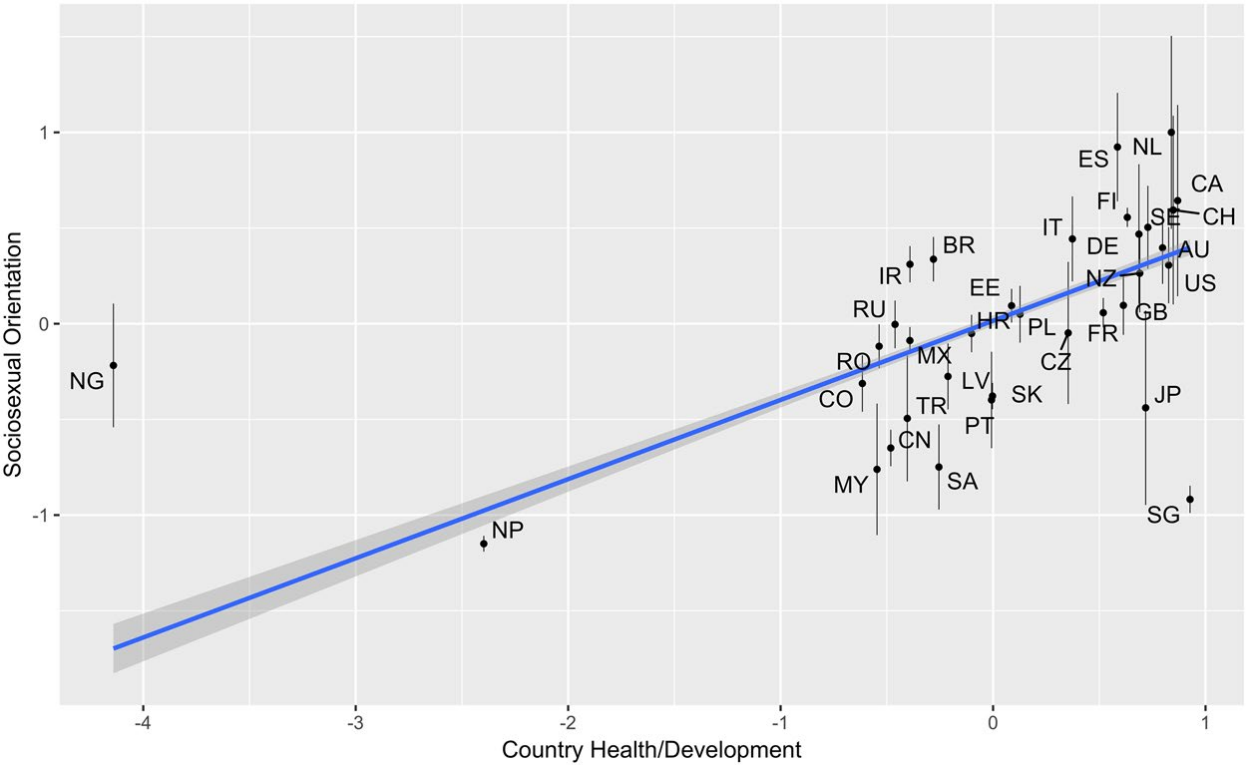
The association between country health/development and sociosexual orientation. Points represent the mean SOI score for individuals in each country, with bars around points representing the 95% confidence interval of the mean. The regression line is the regression line between sociosexual orientation and country health/development. The shaded areas around the regression line are 95% confidence intervals. The country abbreviations in the figure are as follows: AU = Australia; BR = Brazil; CA = Canada; CH = Switzerland; CN = China; CO = Colombia; CZ = Czechia; DE = Germany; EE = Estonia; ES = Spain; FI = Finland; FR = France; GB = United Kingdom of Great Britain and Northern Ireland; HR = Croatia; IR = Iran; IT = Italy; JP = Japan; MX = Mexico; MY = Malaysia; NG = Nigeria; NL = Netherlands; NP = Nepal; NZ = New Zealand; LV = Latvia; PL = Poland; PT = Portugal; RO = Romania; RU = Russia; SA = Saudi Arabia; SE = Sweden; SG = Singapore; SK = Slovakia; TR = Turkey; US = United States of America.

The fixed effects for the model predicting participant SOI are reported in Table 3. The intercept was negative and significant, suggesting that when taking our sample as a whole, women tended to have a restricted sociosexual orientations. There was a significant association between participants’ age and participant SOI, such that older women were more likely to have an unrestricted sociosexuality. There was also a significant, positive association between country Health/Development, such that the greater the health and/or development of a nation, the more likely women were to have an unrestricted sociosexual orientation (Fig. 2). There was no significant effect of the country Inequality factor.

**Table 3:** The fixed effects for the model predicting participant SOI.

	Estimate (Std. Error)	*t*-value (approx.. *df*)	*p*-value
Intercept	−0.60 (0.17)	−3.61 (4.50)	0.018
Participant Age	0.02 (0.004)	5.33 (1.70)	0.047
Country Health/Development Factor	0.25 (0.08)	2.92 (15.72)	0.010
Country Inequality Factor	0.07 (0.10)	0.69 (8.20)	0.510

## Discussion

We found that women’s facial masculinity preferences varied cross-culturally in association with national health and developmental indices. This factor included demographic indices of economic development, such as Human Developmental Indices (HDI) and urbanization. Women living in countries with high HDI and greater urbanization stated stronger preferences for facial masculinity than women from countries with lower HDIs. This finding corroborates those from previous research that reported among 12 cultures that HDI was positively associated with women’s preferences for facial masculinity^41^ and adds to a growing body of research demonstrating that urbanization is positively associated with women’s preferences for masculine facial features^40^. Economic development and access to Western media has been shown to predict greater facial masculinity preferences among El Salvadorian women with access to the Internet compared to women without Internet access^85^. In a related line of research, varying the frequency of secondary sexual traits can generate directional preferences for body shape^86^ and facial hair^42^. Thus, the results from the present study, which span a larger sample of countires and bigger sample size of respondents, lend support to the theory that greater economic development can drive preferences for potentially more distinctive looking mates with better developed secondary sexual facial traits.

Previous cross-cultural research reported that women from countries with low health indices^33^, high pathogens^35^ and high income inequality^38^ stated the strongest preferences for facial masculinity. However, we found the reverse association, such that women’s preferences for facial masculinity were stronger in countries with higher health indices, lower pathogens and greater indices of human economic and social development. Thus, our findings do not support the hypothesis that women’s preferences for facial masculinity reflect facultative trade-offs between paternal investment and indirect genetic benefits associated with testosterone-dependent secondary sexual facial traits^33^. This, in part, could be because country development and health are so highly correlated that they are difficult to disentangle, therefore, the positive effect of country development may mask any predicted, opposite effect of health. Further, the statistical approaches employed in previous studies whereby demographic data and women’s preferences for facial masculinity were aggregated at the national level, may not accurately capture variation in individual preferences in relation to social, ecological and economic factors^63^. In a similar vein, we did not support past cross-cultural analyses reporting that income inequality and homicide rates were strong predictors of women’s preferences for facial masculinity^38^, as we did not find statistically significant associations between measures of violence and women’s facial masculinity preferences across cultures. As the Brooks *et al*.^38^ study was a re-analysis of the data from DeBruine *et al*.^33^, the results could also have emerged due to overestimating the effects of nationally aggregated data on individual preferences^63^. Additional cross-cultural research using mixed effects modelling to characterise cross-cultural variation in women’s preferences for masculinity in men would therefore be valuable.

In support of past cross-cultural research, we found that people living in countries with high health indices, low pathogens and high economic development reported more open sociosexual orientations^56–58^. We also found a significant positive relationship between women’s responses to the Sociosexual Orientation Inventory Revised (SOIR) and facial masculinity preferences, such that sociosexually unrestricted women preferred more facially masculine men than more sociosexually restricted women. As masculine men may incur costs as long-term partners, mating strategies theories propose that women trade-off paternal investment against biological qualities for short-term relationships^32^. However, we note that there were no associations between women’s facial masculinity preferences and levels of social or economic inequality. Thus, our results again do not follow the patterns suggesting facultative trade-offs in preferences under conditions of low health^33^ or high inequality^38^. Instead, women reporting greater willingness to engage in short-term and less romantically committed relationships were more likely to select masculine faces as most sexually attractive under more favourable prevailing environmental and economic conditions. Facially masculine men report greater interest in short-term relationships and engage in more short-term relationships than less facially masculine men^28,29^. While this presents costs in terms of reduced paternal investment, our findings suggest that when social and ecological conditions are more favourable, women high in sexual openness who report greater acceptance of short-term and less romantically committed relationships are potentially better able to realise preferences for more masculinise partners.

It is worth noting that many of the countries in our sample represent so-called WEIRD (Western, educated, industrialized, rich, and democratic) participants^87^. Scott *et al*.^41^ included data from participants living in small-scale societies with less developed market-based economies and found pronounced variation between more traditional subsistence societies and judgments of male aggressiveness. Thus, masculinity was judged as looking more aggressive by participants from countries with greater urban development. Recently, Borras-Guevara *et al*.^30^ reported among a sample of Columbian women that participants with a greater fear of crime and violence stated lower facial masculinity preferences, possibly because masculine facial traits are associated with perceptions of male aggressiveness cross-culturally^41,88–90^. An additional limitation to our study concerns the potential for individual variation in women’s hormone levels to have contributed to their facial masculinity preferences. Thus, women’s mate preferences for markers of biological quality are argued to become stronger at the peri-ovulatory phase of the menstrual cycle^32^. While we had collected information on the menstrual cycle, we collected self-reported data on women’s recollected days since the onset of their last menstrual bleeding rather than hormone measures. These methods are no longer considered to be appropriate in studies of human mate choice as methodological studies have shown they do not accurately characterize current fertility^45^ and simulations have shown they are unreliable^46^. Further, Gangestad *et al*.^46^ noted that counting approaches that have not been verified using hormonal measures require at least 1213 participants for 80% power to detect a medium effect size of *d* = 0.5. Although we have a large cross-cultural sample size, we do not have sufficient power in each sample to test whether within-country fertility influences cross-cultural variation in facial masculinity preferences. Future research may benefit from collecting endocrine data from participants to ascertain current fecundability, which may explain some variation in women’s preferences for male facial masculinity.

In conclusion, our results from a large cross-cultural sample demonstrate that women’s preferences for male facial masculinity are positively associated with economic development and individual differences in sexual openness, which complements findings from cross-cultural studies of men’s preferences for women’s facial femininity^67^. However, we found no evidence that indices of male-male competition (i.e. homicide rates and income inequality) were predictors of women’s facial masculinity preferences. Future cross-cultural research quantifying women’s mate preferences for facial masculinity that include individual differences data among participants from small-scale to more urban settings regarding their fear of violence would be valuable^30^. For the present, our findings suggest that in countries with more favourable social, ecological and economic conditions, wherein any costs of selecting less paternally investing masculine partners may be reduced, women’s preferences for facial masculinity are higher.

## Supplementary information

Electronic Supplementary Materiel

## Data Availability

All data are available from the first author upon request.
