# Supplementary material for: Women’s preferences for men’s facial masculinity are strongest under favorable ecological conditions
Source: Sci Rep. 2019 Mar 4;9:3387. doi: 10.1038/s41598-019-39350-8 (PMC6399235; doi:10.1038/s41598-019-39350-8)
Supplement: Supplementary file 1 — Electronic Supplementary Materiel [file 41598_2019_39350_MOESM1_ESM.pdf]

Supplementary Information

Women's preferences for men's facial masculinity are strongest under favorable ecological conditions.

Urszula M. Marcinkowska[1,2] Markus J Rantala[2] · Anthony J. Lee[3] · Mikhail V. Kozlov[2] · Toivo Aavik[4] · Huajian Cai[5] · Jorge Contreras-Garduño[6] · Oana A. David[7] · Gwenaël Kaminski[8, 9] · Norman P. Li[10] · Ike E. Onyishi[11] · Keshav Prasai[12] · Farid Pazhoohi[13] · Pavol Prokop[14,15] · Sandra L. Rosales Cardozo[16] · Nicolle Sydney[17] ·Hirokazu Taniguchi[18] · Indrikis Krams[19,20,21]· Barnaby J. W. Dixson[22]

Cross-Cultural Masculinity Preferences

Anthony J. Lee: anthony.lee@glasgow.ac.uk (mailto:anthony.lee@glasgow.ac.uk)

- Housekeeping
  - Load Packages and Custom Functions
- Data Preparation
  - Participant Data
  - Trial Data
  - Country Data
    - Collect Country-Level Statistics
    - Proportion of Population Urbanised
    - Data Preparation
    - Conduct Independent Factors Analysis
- Descriptives
  - Participant Statistics
  - Country Statistics
- Conduct Analysis
  - Full Masculinity Model
    - Masculinity Model excluding NP & NG
    - Masculinity Model without SOI
  - SOI Model
  - Relationship Status
  - Plot Data - Masculinity Preference
  - Plot Data - SOI

Housekeeping

Load Packages and Custom Functions

```
require(tidyverse)
require(lme4)
require(lmerTest)
require(psych)
require(ggrepel)

#Easy Standardising Function
z <- function(x,remove.outliers = FALSE,windsorise = FALSE){
    out <- (x - mean(x,na.rm = TRUE))/sd(x,na.rm = TRUE)
    if (remove.outliers == TRUE){
      out <- ifelse(out >3,NA,ifelse(out < -3,NA,out))
```

```
    }
    if (windsorise == TRUE){
      out <- ifelse(out > 3,3,ifelse(out < -3,-3,out))
    }
    return(out)
}

#Descriptives Statistics Function
descriptives <- function(data,variables){
    output <- data.frame("variable"=variables,"N"=NA,"min"=NA,"max"=NA,"mean"=NA,"sd"
=NA)
    d <- data[variables]
    for (v in 1:NROW(variables)){
        output[v,"N"] <- sum(!is.na(d[v]))
        output[v,"min"] <- min(d[,v],na.rm=TRUE)
        output[v,"max"] <- max(d[,v],na.rm=TRUE)
        output[v,"mean"] <- mean(d[,v],na.rm=TRUE)
        output[v,"sd"] <- sd(d[,v],na.rm=TRUE)
    }
    output
}

#Recode Stimuli Sex
rct <- function(.in){
    out <- rep(NA,NROW(.in))
    out[(grep("f50",.in))] <- 0
    out[(grep("m50",.in))] <- 1
    out
}

#Recode SOI
rcsoi <- function(.in){
    suppressWarnings(out <- recode(.in, "Never" = 1,
                        "Very seldom" = 2,
                        "About once every 2 or 3 months" = 3,
                        "About once a month" = 4,
                        "About once every 2 or 3 weeks" = 5,
                        "About once a week" = 6,
                        "Several times a week" = 7,
                        "Nearly every day" = 8,
                        "At least once a day" = 9,
                        "0" = 1,
                        "1" = 2,
                        "2" = 3,
                        "3" = 4,
                        "4" = 5,
                        "5-6" = 6,
```

```
                        "7-9" = 7,
                        "10-19" = 8,
                        "More than 20" = 9) %>%
        as.numeric())
    out
}

#Recode Country Names
rccountry <- function(.in){
out <- recode(.in,  "Iran (Islamic Republic of)" = "Iran, Islamic Republic of...",
            "Czechia" = "Czech Republic",
            "Slovak Republic" = "Slovakia",
            "United States" = "United States of America",
            "United Kingdom" = "United Kingdom of Great Britain and Northern Ireland"
,
            "Iran, Islamic Rep." = "Iran, Islamic Republic of...",
            "Russia" = "Russian Federation",
            "Iran" = "Iran, Islamic Republic of...")
 out
}
```

Data Preparation

Participant Data

Prepare data for analysis. Commented out N values refers to number of participants remaining after the filter on that line is applied.

```
data <- read.csv("MasculinityForBarnaby 08.08.17.csv",
          stringsAsFactors = FALSE) %>% #N = 7739
    filter(Age != "less than 17") %>%   #N = 7638
    mutate(Age = as.numeric(Age)) %>%
    filter(Age <= 40) %>%   #N = 6939
    filter(Orientation == "Exclusively heterosexual") %>%   #N = 5429
    group_by(Country) %>%
    mutate(n = n()) %>%
    ungroup() %>%
    dplyr::select(ID,n,Sex,Age,Relationship,Country,X1:X20,X.21:X.29) %>%
    rename( t1 = X1,t2 = X2,t3 = X3,t4 = X4,t5 = X5,
            t6 = X6,t7 = X7,t8 = X8,t9 = X9,t10 = X10,
                t11 = X11,t12 = X12,t13 = X13,t14 = X14,t15 = X15,
                t16 = X16,t17 = X17,t18 = X18,t19 = X19,t20 = X20,
                soi1 = X.21,soi2 = X.22,soi3 = X.23,
                soi4 = X.24,soi5 = X.25,soi6 = X.26,
                soi7 = X.27,soi8 = X.28,soi9 = X.29) %>%
    mutate( t1 = rct(t1),t2 = rct(t2),t3 = rct(t3),
            t4 = rct(t4),t5 = rct(t5),t6 = rct(t6),
            t7 = rct(t7),t8 = rct(t8),t9 = rct(t9),
            t10 = rct(t10),t11 = rct(t11),t12 = rct(t12),
            t13 = rct(t13),t14 = rct(t14),t15 = rct(t15),
            t16 = rct(t16),t17 = rct(t17),t18 = rct(t18),
            t19 = rct(t19),t20 = rct(t20),
                soi1 = rcsoi(soi1),soi2 = rcsoi(soi2),soi3 = rcsoi(soi3),
                soi7 = rcsoi(soi7), soi8 = rcsoi(soi8), soi9 = rcsoi(soi9)) %>%
    mutate(soit = as.numeric(soi1) + as.numeric(soi2) + as.numeric(soi3) +
                  soi4 + soi5 + ((soi6*-1) + 10) +
                  soi7 + soi8 + soi9) %>%
    mutate(zAge = z(Age),zsoit = z(soit)) %>%
    filter(!is.na(soit)) %>%    #N = 4572
    filter(n >= 10) #N = 4483
```

```
## Warning: package 'bindrcpp' was built under R version 3.4.4
```

Trial Data

```
data <- gather(data,key = "trial_id",value = "dv", t1:t20)
```

Country Data

```
country.data <- data.frame(Country = unique(data$Country),stringsAsFactors = FALSE)
```

Collect Country-Level Statistics

Region

Geographical region taken from The World Bank's "Country and Lendings Groups" Classifications.

```
region <- read.csv("World Bank Regions.csv",stringsAsFactors = FALSE,skip = 5) %>%
    dplyr::select(x.2,x.5) %>%
  rename(Country = x.2, region = x.5) %>%
  mutate(Country = rccountry(Country))

country.data <- left_join(country.data,region,by = "Country")
```

Fertility Rate

Fertility Rate taken from The World Bank's Database.

```
fertility <- read.csv("API_SP.DYN.TFRT.IN_DS2_en_csv_v2.csv",stringsAsFactors = FALSE
,skip = 4) %>%
    rename(Country = Country.Name,fertility = X2012) %>%
    dplyr::select(Country,fertility) %>%
    mutate( Country = rccountry(Country))

 country.data <- left_join(country.data,fertility, by = "Country")
```

Years Lost due to Communicable Disease

From The World Health Organisation Database (2010 data)

```
YLL <- read.csv("GHE2015_YLL-2010-country.csv",stringsAsFactors = FALSE,skip = 6,blan
k.lines.skip = TRUE,header = FALSE)[c(1,5,6),] %>%
    dplyr::select(V4,V8:V190) %>%
    mutate(V4 = recode(V4,  "GHE cause" = "Country",
                            "All Causes" = "all_causes",
                            "Communicable, maternal, perinatal and nutritional condit
ions" = "disease")) %>%
    column_to_rownames("V4") %>%
    t() %>%
    as.data.frame(stringsAsFactors = FALSE) %>%
    mutate( all_causes = as.numeric(all_causes),
            disease = as.numeric(disease),
            yltd = disease/all_causes,
            Country = rccountry(Country)) %>%
            dplyr::select(Country,yltd)

country.data <- left_join(country.data,YLL,by = "Country")
```

Historical Disease Prevalence

From Murray & Schaller (2010)

```
pathogen <- read.csv("Historical_Pathogen_Prevalence.csv",stringsAsFactors = FALSE) %
>%
    rename(Country = Nation) %>%
    filter(Country != "United Kingdom") %>%
    mutate( Country = ifelse(Country == "England","United Kingdom of Great Britain an
d Northern Ireland",Country),
            Country = rccountry(Country))

country.data <- left_join(country.data,pathogen,by = "Country")
```

Homicide Rate

From The World Bank's Database.

```
homicide <- read.csv("API_VC.IHR.PSRC.P5_DS2_en_csv_v2.csv",stringsAsFactors = FALSE,
skip = 4) %>%
    rename(Country = Country.Name,homicide = X2012) %>%
    mutate( Country = rccountry(Country),
            homicide = ifelse(is.na(homicide),X2010,homicide),
            homicide = ifelse(is.na(homicide),X2014,homicide)) %>%
    dplyr::select(Country,homicide)

country.data <- left_join(country.data,homicide, by = "Country")
```

Proportion of Population Urbanised

From The World Bank's Database.

```
urban <- read.csv("API_SP.URB.TOTL.IN.ZS_DS2_en_csv_v2.csv",stringsAsFactors = FALSE,
skip = 4) %>%
    rename(Country = Country.Name,urban = X2012) %>%
    dplyr::select(Country,urban) %>%
    mutate(Country = rccountry(Country))

country.data <- left_join(country.data,urban, by = "Country")
```

GDP

From The World Bank's Database.

```
gdp <- read.csv("API_NY.GDP.MKTP.CD_DS2_en_csv_v2.csv",stringsAsFactors = FALSE, skip
= 4) %>%
    rename(Country = Country.Name,gdp = X2012) %>%
    dplyr::select(Country,gdp) %>%
    mutate(Country = rccountry(Country))

country.data <- left_join(country.data,gdp,by = "Country")
```

HDI

From The United Nations Database.

```
hdi <- read.csv("2016_Statistical_Annex_Table_2.csv",stringsAsFactors = FALSE, blank.
lines.skip = TRUE,skip =4) %>%
    rename(Country = X.1,hdi = X2012) %>%
    dplyr::select(Country, hdi) %>%
    filter(!is.na(Country)) %>%
    mutate( Country = rccountry(Country),
            hdi = as.numeric(hdi))

country.data <- left_join(country.data,hdi, by = "Country")
```

GINI

From The World Bank's Database.

```
gini <- read.csv("API_SI.POV.GINI_DS2_en_csv_v2.csv",stringsAsFactors = FALSE, skip =
4) %>%
    rename(Country = Country.Name,gini = X2012) %>%
    mutate( Country = rccountry(Country),
            gini = ifelse(is.na(gini),X2011,gini),
            gini = ifelse(is.na(gini),X2013,gini),
            gini = ifelse(is.na(gini),X2010,gini),
            gini = ifelse(is.na(gini),X2014,gini),
            gini = ifelse(is.na(gini),X2009,gini),
            gini = ifelse(is.na(gini),X2015,gini),
            gini = ifelse(is.na(gini),X2008,gini)) %>%
    dplyr::select(Country,gini)

country.data <- left_join(country.data,gini,by = "Country")
```

GII

From The United Nations Database (2015 data).

```
gii <- read.csv("2016_Statistical_Annex_Table_5.csv",stringsAsFactors = FALSE, blank.
lines.skip = TRUE,skip =2) %>%
    rename(Country = X.1,gii = Gender.Inequality.Index) %>%
    dplyr::select(Country, gii) %>%
    filter(!is.na(Country)) %>%
    mutate( Country = rccountry(Country),
            gii = as.numeric(gii))

country.data <- left_join(country.data,gii,by = "Country")
```

Mortality Rates

From The World Bank's Database.

```
mortality <- read.csv("API_SP.DYN.CDRT.IN_DS2_en_csv_v2.csv",stringsAsFactors = FALSE
, skip = 4) %>%
    rename(Country = Country.Name,mortality = X2012) %>%
    dplyr::select(Country,mortality) %>%
    mutate(Country = rccountry(Country))

country.data <- left_join(country.data,mortality,by = "Country")
```

Adult Life Expectancy

From The World Health Organisation's Database.

```
life.exp <- read.csv("WHOSIS_000001,WHOSIS_000015.csv",stringsAsFactors = FALSE,skip=
1) %>%
    dplyr::select(Country,Year,X.Both.sexes) %>%
  rename(year = Year,life.exp = X.Both.sexes) %>%
  mutate(year = as.numeric(year)) %>%
  filter(year == 2012) %>%
  dplyr::select(Country,life.exp) %>%
  mutate(Country = rccountry(Country))

country.data <- left_join(country.data,life.exp,by = "Country")
```

Data Preparation

Standardise each country-level statistic and replace missing statistics with the mean for all countries.

```
country.data <- mutate( country.data,   fertility = z(fertility),
                                        yltd = z(yltd),
                                        pathogen9 = z(pathogen9),
                                        pathogen7 = z(pathogen7),
                                        homicide = z(homicide),
                                        urban = z(urban),
                                        gdp = z(gdp),
                                        hdi = z(hdi),
                                        gini = z(gini),
                                        gii = z(gii),
                                        mortality = z(mortality),
                                        life.exp = z(life.exp))

country.data[is.na(country.data)] <- 0
```

Conduct Independent Factors Analysis

```
d <- dplyr::select(country.data,fertility,yltd,pathogen9,homicide,urban,gdp,hdi,gini,
gii,mortality,life.exp)

country.fa <- principal(d,nfactors = 2,rotate = "oblimin")

country.fa.loadings <- country.fa$loadings[1:11,1:2] %>%
    as.data.frame(row.names = row.names(.)) %>%
    rownames_to_column(var = "statistic") %>%
    arrange(desc(abs(TC1)))

country.fa.loadings
```

```
##    statistic        TC1           TC2
## 1   life.exp -0.9491521 -0.0006044123
## 2        hdi -0.9055066 -0.1864010956
## 3       yltd  0.8721456  0.1111766660
## 4      urban -0.8187675  0.3685915259
## 5  fertility  0.7605397  0.0318030290
## 6  pathogen9  0.5101188  0.3563879882
## 7        gii  0.4090867  0.5615986245
## 8  mortality  0.3434875 -0.7409053914
## 9        gdp -0.2653001  0.2603918752
## 10  homicide  0.1594108  0.7314636960
## 11      gini  0.1483109  0.8809988022
```

Factor 1 appears to represent Health/Development, while Factor 2 appears to encompass country statistics associated with Inequality. Factor scores are reversed-scored so that positive associations with facial masculinity preferences are predicted with both.

```
country.fa.scores <- country.fa$scores%>%
    as.data.frame() %>%
    rename(health.fa = TC1, inequality.fa = TC2) %>%
    mutate(health.fa = z(health.fa * -1),
           inequality.fa = z(inequality.fa * -1))

country.data <- cbind(country.data,country.fa.scores)

cor.test(country.data$health.fa,country.data$inequality.fa)
```

```
##
##  Pearson's product-moment correlation
##
## data:  country.data$health.fa and country.data$inequality.fa
## t = 1.5032, df = 32, p-value = 0.1426
## alternative hypothesis: true correlation is not equal to 0
## 95 percent confidence interval:
##  -0.08908937  0.54743665
## sample estimates:
##       cor
## 0.2568133
```

Descriptives

Participant Statistics

```
analysis.data <- left_join(data,country.data,by = "Country")

participant.data <- analysis.data %>%
    group_by(ID) %>%
    filter(row_number() == 1) %>%
    ungroup() %>% as.data.frame()

descriptives(participant.data,c("Age","soit"))
```

```
##   variable    N min max     mean        sd
## 1      Age 4483  18  40 25.20857  5.437692
## 2     soit 4483   9  78 27.92059 13.554110
```

```
table(participant.data$Relationship)
```

```
##
##                   Difficult to say                No              Yes
##                16               356              1625             2486
```

```
analysis.data <- mutate(analysis.data,Age = zAge,
                        soit = zsoit,
                        Relationship = as.numeric(recode(Relationship, "Yes" = ".5",
                                              "No" = "-.5",
                                              "Difficult to say" = "NA")))
```

Country Statistics

```
country.descriptives <- participant.data %>%
  group_by(Country) %>%
  summarise(N = n()) %>%
  left_join(select(country.data,Country,health.fa,inequality.fa),by = "Country")

country.descriptives
```

```
## # A tibble: 34 x 4
##    Country            N health.fa inequality.fa
##    <chr>          <int>     <dbl>         <dbl>
##  1 Australia         68    0.798        -0.0711
##  2 Brazil           218   -0.281        -2.49
##  3 Canada             9    0.869         0.305
##  4 China             77   -0.481        -0.622
##  5 Colombia          68   -0.615        -2.65
##  6 Croatia          173   -0.101         0.977
##  7 Czech Republic    10    0.354         1.06
##  8 Estonia          245    0.0872        0.748
##  9 Finland         1066    0.632         0.894
## 10 France           301    0.519         0.355
## # ... with 24 more rows
```

Conduct Analysis

Full Masculinity Model

```
model.fa <- glmer(  dv ~ Age + soit + health.fa + inequality.fa +
                     (1 | ID) +
                      (0 + health.fa + inequality.fa | ID) +
                     (1 | trial_id) +
                      (0 + Age + soit + health.fa + inequality.fa | trial_id) +
                     (1 | region/Country) +
                      (0 + Age + soit | region/Country),
        data = analysis.data,family = "binomial")
```

```
summary(model.fa)
```

```
## Generalized linear mixed model fit by maximum likelihood (Laplace
##   Approximation) [glmerMod]
##  Family: binomial  ( logit )
## Formula: dv ~ Age + soit + health.fa + inequality.fa + (1 | ID) + (0 +
##     health.fa + inequality.fa | ID) + (1 | trial_id) + (0 + Age +
##     soit + health.fa + inequality.fa | trial_id) + (1 | region/Country) +
##     (0 + Age + soit | region/Country)
##    Data: analysis.data
## Control:
## glmerControl(optimizer = "bobyqa", optCtrl = list(maxfun = 10000))
##
##      AIC      BIC   logLik deviance df.resid
##  97764.9  98028.2 -48854.5  97708.9    89632
##
## Scaled residuals:
##     Min      1Q  Median      3Q     Max
## -8.4538 -0.6449  0.2102  0.6317  7.1447
##
## Random effects:
##  Groups           Name          Variance  Std.Dev.  Corr
##  ID               health.fa     1.204e-06 0.0010971
##                   inequality.fa 3.944e-04 0.0198603 0.83
##  ID.1             (Intercept)   1.541e+00 1.2413176
##  Country.region   Age           2.686e-05 0.0051827
##                   soit          1.124e-04 0.0105999 1.00
##  Country.region.1 (Intercept)   1.870e-02 0.1367644
##  trial_id         Age           2.564e-04 0.0160139
##                   soit          4.949e-03 0.0703513 -0.40
##                   health.fa     7.096e-02 0.2663822 -0.45  0.59
##                   inequality.fa 8.620e-03 0.0928428  0.45 -0.31 -0.23
##  trial_id.1       (Intercept)   1.576e+00 1.2552051
##  region           Age           0.000e+00 0.0000000
##                   soit          1.209e-08 0.0001099  NaN
##  region.1         (Intercept)   6.124e-01 0.7825713
```

```
## Number of obs: 89660, groups:
## ID, 4483; Country:region, 34; trial_id, 20; region, 7
##
## Fixed effects:
##                  Estimate Std. Error z value Pr(>|z|)
## (Intercept)    -0.494058   0.454793  -1.086 0.277330
## Age             0.036003   0.005796   6.211 5.25e-10 ***
## soit            0.111218   0.028866   3.853 0.000117 ***
## health.fa       0.286266   0.142668   2.007 0.044801 *
## inequality.fa -0.047820   0.120921  -0.395 0.692498
## ---
## Signif. codes:  0 '***' 0.001 '**' 0.01 '*' 0.05 '.' 0.1 ' ' 1
##
## Correlation of Fixed Effects:
##             (Intr) Age    soit   hlth.f
## Age         -0.197
## soit         0.015 -0.195
## health.fa    0.240 -0.136  0.083
## inequalty.f  0.075  0.107 -0.032 -0.178
## convergence code: 1
## Model failed to converge with max|grad| = 2.22036 (tol = 0.001, component 1)
```

Masculinity Model excluding NP & NG

```
model.fa1 <- glmer( dv ~ Age + soit + health.fa + inequality.fa +
                     (1 | ID) +
                      (0 + health.fa + inequality.fa | ID) +
                     (1 | trial_id) +
                      (0 + Age + soit + health.fa + inequality.fa | trial_id) +
                     (1 | region/Country) +
                      (0 + Age + soit | region/Country),
                data = filter(analysis.data,Country != "Nepal" | Country != "Nigeria"
),family = "binomial")
```

```
summary(model.fa1)
```

```
## Generalized linear mixed model fit by maximum likelihood (Laplace
##   Approximation) [glmerMod]
##  Family: binomial  ( logit )
## Formula: dv ~ Age + soit + health.fa + inequality.fa + (1 | ID) + (0 +
##     health.fa + inequality.fa | ID) + (1 | trial_id) + (0 + Age +
##     soit + health.fa + inequality.fa | trial_id) + (1 | region/Country) +
##     (0 + Age + soit | region/Country)
##    Data: filter(analysis.data, Country != "Nepal" | Country != "Nigeria")
```

```
## Control:
## glmerControl(optimizer = "bobyqa", optCtrl = list(maxfun = 10000))
##
##      AIC      BIC   logLik deviance df.resid
##  97756.1  98019.4 -48850.1  97700.1    89632
##
## Scaled residuals:
##     Min      1Q  Median      3Q     Max
## -8.4726 -0.6452  0.2102  0.6315  7.2176
##
## Random effects:
##  Groups           Name          Variance  Std.Dev.  Corr
##  ID               health.fa     0.000e+00 0.000e+00
##                   inequality.fa 1.055e-07 3.248e-04  NaN
##  ID.1             (Intercept)   1.542e+00 1.242e+00
##  Country.region   Age           2.086e-10 1.444e-05
##                   soit          1.631e-12 1.277e-06 1.00
##  Country.region.1 (Intercept)   4.212e-02 2.052e-01
##  trial_id         Age           8.545e-03 9.244e-02
##                   soit          5.065e-03 7.117e-02 -0.41
##                   health.fa     7.111e-02 2.667e-01 -0.49  0.59
##                   inequality.fa 8.670e-03 9.311e-02  0.46 -0.31 -0.22
##  trial_id.1       (Intercept)   1.112e+00 1.055e+00
##  region           Age           0.000e+00 0.000e+00
##                   soit          9.751e-15 9.875e-08  NaN
##  region.1         (Intercept)   1.846e-01 4.296e-01
## Number of obs: 89660, groups:
## ID, 4483; Country:region, 34; trial_id, 20; region, 7
##
## Fixed effects:
##               Estimate Std. Error z value Pr(>|z|)
## (Intercept)    0.29471    0.31607   0.932   0.3511
## Age            0.19889    0.03072   6.475 9.46e-11 ***
## soit           0.11278    0.02869   3.931 8.47e-05 ***
## health.fa      0.19995    0.11952   1.673   0.0943 .
## inequality.fa -0.05530    0.11518  -0.480   0.6312
## ---
## Signif. codes:  0 '***' 0.001 '**' 0.01 '*' 0.05 '.' 0.1 ' ' 1
##
## Correlation of Fixed Effects:
##             (Intr) Age    soit   hlth.f
## Age         -0.014
## soit        -0.007 -0.240
## health.fa    0.231 -0.191  0.110
## inequalty.f  0.224  0.060 -0.043  0.019
## convergence code: 0
## Model failed to converge with max|grad| = 0.00172817 (tol = 0.001, component 1)
```

Masculinity Model without SOI

```
model.fa2 <- glmer( dv ~ Age + health.fa + inequality.fa +
                      (1 | ID) +
                        (0 + health.fa + inequality.fa | ID) +
                      (1 | trial_id) +
                        (0 + Age + health.fa + inequality.fa | trial_id) +
                      (1 | region/Country) +
                        (0 + Age | region/Country),
                data = analysis.data,family = "binomial")
```

```
summary(model.fa2)
```

```
## Generalized linear mixed model fit by maximum likelihood (Laplace
##   Approximation) [glmerMod]
##  Family: binomial  ( logit )
## Formula:
## dv ~ Age + health.fa + inequality.fa + (1 | ID) + (0 + health.fa +
##     inequality.fa | ID) + (1 | trial_id) + (0 + Age + health.fa +
##     inequality.fa | trial_id) + (1 | region/Country) + (0 + Age |
##     region/Country)
##    Data: analysis.data
## Control:
## glmerControl(optimizer = "bobyqa", optCtrl = list(maxfun = 10000))
##
##      AIC      BIC   logLik deviance df.resid
##  97803.8  97982.4 -48882.9  97765.8    89641
##
## Scaled residuals:
##     Min      1Q  Median      3Q     Max
## -9.0713 -0.6444  0.2106  0.6322  7.2470
##
## Random effects:
##  Groups           Name          Variance  Std.Dev.  Corr
##  ID               health.fa     1.234e-09 3.513e-05
##                   inequality.fa 1.380e-07 3.715e-04 0.74
##  ID.1             (Intercept)   1.547e+00 1.244e+00
##  Country.region   Age           2.166e-05 4.654e-03
##  Country.region.1 (Intercept)   3.333e-02 1.826e-01
##  trial_id         Age           2.307e-04 1.519e-02
##                   health.fa     8.039e-02 2.835e-01 -0.38
##                   inequality.fa 8.638e-03 9.294e-02  0.44 -0.24
##  trial_id.1       (Intercept)   1.568e+00 1.252e+00
##  region           Age           0.000e+00 0.000e+00
##  region.1         (Intercept)   2.402e-01 4.901e-01
```

```
## Number of obs: 89660, groups:
## ID, 4483; Country:region, 34; trial_id, 20; region, 7
##
## Fixed effects:
##                 Estimate Std. Error z value Pr(>|z|)
## (Intercept)    -0.658126   0.380902  -1.728   0.0840 .
## Age             0.038810   0.005629   6.895  5.4e-12 ***
## health.fa       0.244259   0.128964   1.894   0.0582 .
## inequality.fa -0.051164   0.119905  -0.427   0.6696
## ---
## Signif. codes:  0 '***' 0.001 '**' 0.01 '*' 0.05 '.' 0.1 ' ' 1
##
## Correlation of Fixed Effects:
##             (Intr) Age    hlth.f
## Age         -0.231
## health.fa    0.220 -0.130
## inequalty.f  0.164  0.081 -0.027
## convergence code: 1
## Model failed to converge with max|grad| = 0.00997979 (tol = 0.001, component 1)
```

SOI Model

```
model.soi <- lmer(zsoit ~ Age + health.fa + inequality.fa  +
                      (1 | region/Country) +
                        (0 + Age | region/Country),
                data = participant.data)
```

```
summary(model.soi)
```

```
## Linear mixed model fit by REML. t-tests use Satterthwaite's method [
## lmerModLmerTest]
## Formula: zsoit ~ Age + health.fa + inequality.fa + (1 | region/Country) +
##     (0 + Age | region/Country)
##    Data: participant.data
##
## REML criterion at convergence: 11387.4
##
## Scaled residuals:
##     Min      1Q  Median      3Q     Max
## -2.3431 -0.6622 -0.1557  0.6029  4.5581
##
## Random effects:
##  Groups           Name        Variance  Std.Dev.
##  Country.region   Age         9.046e-05 0.009511
##  Country.region.1 (Intercept) 6.014e-02 0.245242
##  region           Age         1.620e-05 0.004025
##  region.1         (Intercept) 7.149e-02 0.267374
##  Residual                     7.247e-01 0.851274
## Number of obs: 4483, groups:  Country:region, 34; region, 7
##
## Fixed effects:
##                Estimate Std. Error        df t value Pr(>|t|)
## (Intercept)   -0.601725   0.166568  4.498172  -3.612   0.0184 *
## Age            0.023748   0.004453  1.704180   5.333   0.0465 *
## health.fa      0.246797   0.084592 15.715686   2.917   0.0102 *
## inequality.fa  0.071688   0.104018  8.198346   0.689   0.5097
## ---
## Signif. codes:  0 '***' 0.001 '**' 0.01 '*' 0.05 '.' 0.1 ' ' 1
##
## Correlation of Fixed Effects:
##             (Intr) Age    hlth.f
## Age         -0.454
## health.fa    0.169  0.038
## inequalty.f  0.254  0.034 -0.139
```

Relationship Status

```
model.rel <- glmer( dv ~ Relationship + Age + (1 | ID) +
                                                              (1  | trial_id) +
                                                                (0 + Relationship + Age
| trial_id) +
                                                              (1  | Country/region) +
                                                                (0 + Relationship + Age
| Country/region),
                data = analysis.data,family = "binomial")
```

```
summary(model.rel)
```

```
## Generalized linear mixed model fit by maximum likelihood (Laplace
##   Approximation) [glmerMod]
##  Family: binomial  ( logit )
## Formula: 
## dv ~ Relationship + Age + (1 | ID) + (1 | trial_id) + (0 + Relationship +  
##     Age | trial_id) + (1 | Country/region) + (0 + Relationship +  
##     Age | Country/region)
##    Data: analysis.data
## 
##      AIC      BIC   logLik deviance df.resid 
##  90475.8  90624.9 -45221.9  90443.8    82204 
## 
## Scaled residuals: 
##     Min      1Q  Median      3Q     Max 
## -7.4964 -0.6560  0.2264  0.6390  8.3805 
## 
## Random effects:
##  Groups           Name         Variance  Std.Dev.  Corr 
##  ID               (Intercept)  1.479e+00 1.216e+00      
##  Country          Relationship 8.941e-13 9.456e-07      
##                   Age          3.955e-10 1.989e-05 0.38 
##  region.Country   Relationship 8.289e-10 2.879e-05      
##                   Age          1.266e-11 3.559e-06 -1.00
##  Country.1        (Intercept)  1.392e-01 3.730e-01      
##  region.Country.1 (Intercept)  2.210e-03 4.701e-02      
##  trial_id         Relationship 3.169e-03 5.629e-02      
##                   Age          6.368e-03 7.980e-02 -0.27
##  trial_id.1       (Intercept)  1.046e+00 1.023e+00      
## Number of obs: 82220, groups:  
## ID, 4111; Country, 34; region:Country, 34; trial_id, 20
## 
## Fixed effects:
##              Estimate Std. Error z value Pr(>|z|)    
## (Intercept)   0.16410    0.23890   0.687    0.492    
## Relationship -0.02001    0.04550  -0.440    0.660    
## Age           0.20286    0.02952   6.873 6.29e-12 ***
## ---
## Signif. codes:  0 '***' 0.001 '**' 0.01 '*' 0.05 '.' 0.1 ' ' 1
## 
## Correlation of Fixed Effects:
##             (Intr) Rltnsh
## Relationshp -0.021       
## Age         -0.001 -0.149
```

Plot Data - Masculinity Preference

```
country.code <- read.delim("Country Codes Edit.txt",sep=",",col.names = c("resident",
"Nation"),header = FALSE,stringsAsFactors = FALSE) %>%
  rename(Country = Nation,country = resident) %>%
  mutate(Country = recode(Country,"Russia" = "Russian Federation","Iran (Islamic Repu
blic of)" = "Iran, Islamic Republic of...","Czechia" = "Czech Republic"))

plot.data <- group_by(analysis.data,ID) %>%
    summarise(dv.m = mean(dv,na.rm = TRUE),
              soit.m = mean(soit,na.rm = TRUE)) %>%
    ungroup() %>%
    left_join(filter(group_by(analysis.data,ID),row_number() == 1),by = "ID") %>%
    dplyr::select(ID,dv.m,soit.m,health.fa,Country) %>%
  left_join(country.code,by = "Country")
plot.data2 <- group_by(plot.data, Country) %>%
    summarise(dv.c = mean(dv.m,na.rm = TRUE),
            dv.min = mean(dv.m,na.rm = TRUE) - qnorm(0.95)*sd(dv.m,na.rm = TRUE)/sqrt
(n()),
            dv.max = mean(dv.m,na.rm = TRUE) + qnorm(0.95)*sd(dv.m,na.rm = TRUE)/sqrt
(n()),
              soit.c = mean(soit.m,na.rm = TRUE),
            soit.min = mean(soit.m,na.rm = TRUE) - qnorm(0.95)*sd(soit.m,na.rm = TRUE
)/sqrt(n()),
            soit.max = mean(soit.m,na.rm = TRUE) + qnorm(0.95)*sd(soit.m,na.rm = TRUE
)/sqrt(n())) %>%
    left_join(filter(group_by(plot.data,Country),row_number() == 1), by = "Country")
%>%
  mutate(label = row_number())

ggplot(data = plot.data2,aes(y = dv.c,x = health.fa)) +
    geom_pointrange(aes(ymin = dv.min, ymax = dv.max),shape=20,size=.2) +
    geom_smooth(data = plot.data,aes(y = dv.m,x = health.fa),method = lm, formula = y
~ x,se = TRUE) +
    theme(legend.position = "none") +
    ylab("Proportion of trials the masculinised face was chosen.") +
    xlab("Country Health/Development") +
    geom_text_repel(aes(label = country))
```

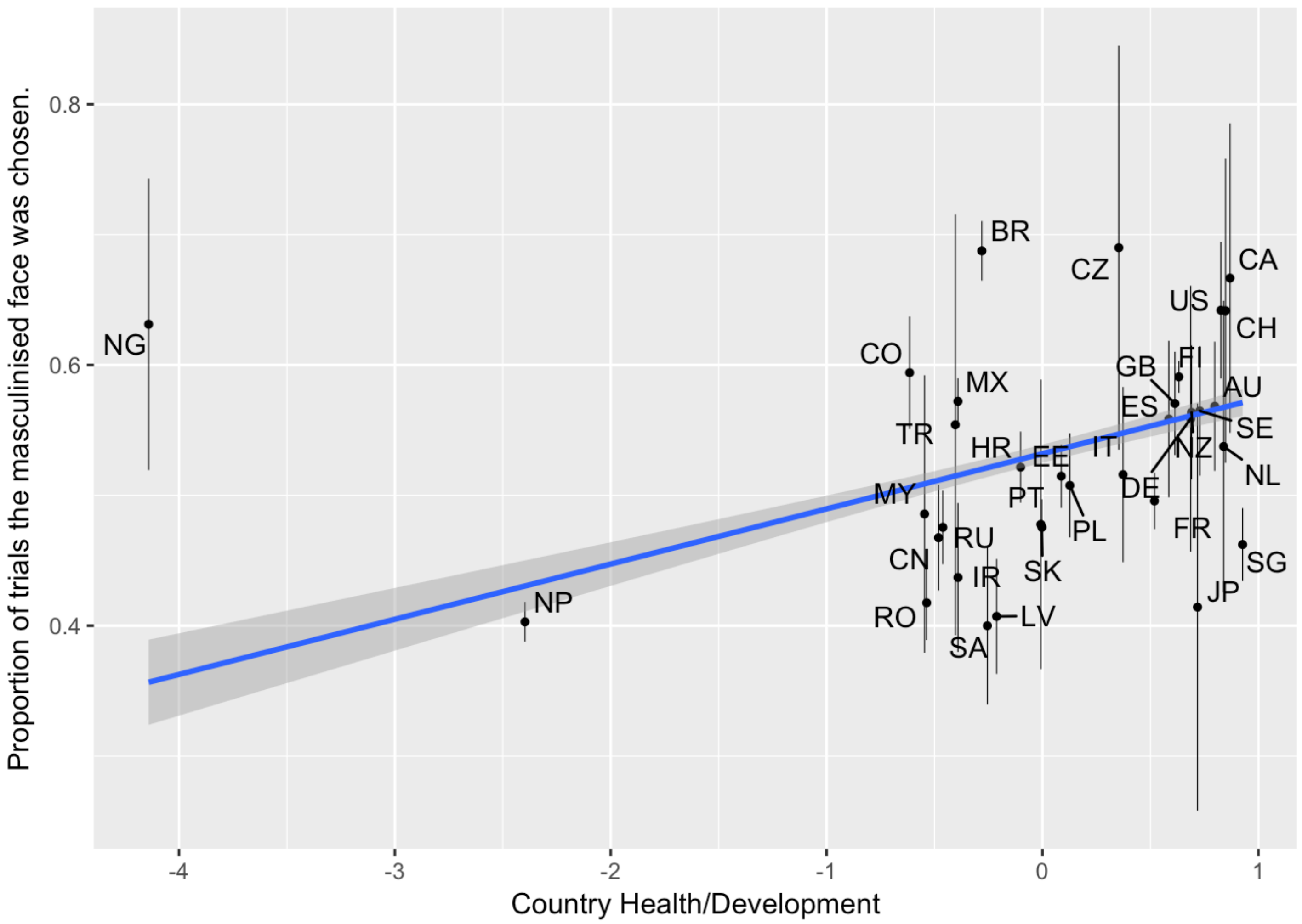


```
#ggsave("Plot_MascPref.png")
```

Plot Data - SOI

```
ggplot(data = plot.data2,aes(y = soit.c,x = health.fa)) +
    geom_pointrange(aes(ymin = soit.min, ymax = soit.max),shape=20,size=.2) +
    geom_smooth(data = plot.data, aes(y = soit.m),method = lm, formula = y ~ x,se = T
RUE) +
    theme(legend.position = "none") +
    ylab("Sociosexual Orientation") +
    xlab("Country Health/Development") +
    geom_text_repel(aes(label = country))
```

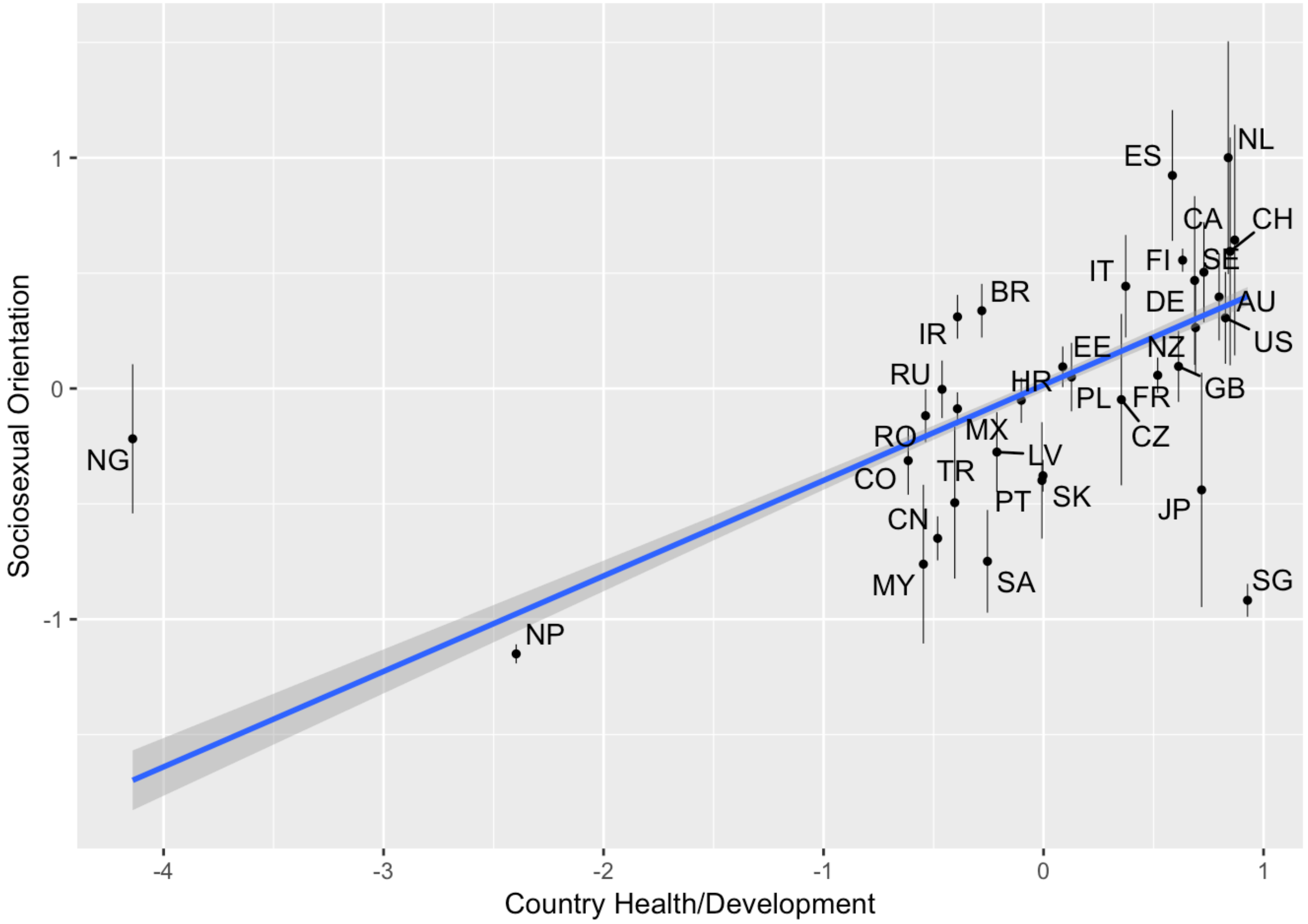


```
#ggsave("Plot_SOI.png")
```
